# Appendiceal Metastasis of Breast Cancer: A Case Report and a Literature Review

**DOI:** 10.7759/cureus.57929

**Published:** 2024-04-09

**Authors:** Takuki Yagyu, Hisanori Miki, Yuichiro Kikawa, Toshinori Kobayashi, Mitsugu Sekimoto

**Affiliations:** 1 Department of Surgery, Kansai Medical University, Hirakata, JPN

**Keywords:** laparoscopic surgery, invasive lobular carcinoma, metastasis, appendix, breast cancer

## Abstract

Appendiceal metastases of breast cancer (BC) are very rare, and there are few reports of resection. Asymptomatic appendiceal enlargement is often suspected to be a primary appendiceal tumor, making it difficult to suspect metastatic tumors, especially metastases from BC. On the other hand, advances in drug therapy, including hormonal therapy for BC, have prolonged survival, and there is a possibility of encountering metastatic cases that have rarely been seen before. We herein present a case in which an enlarged appendix, identified during hormonal therapy for advanced BC, was laparoscopically removed and diagnosed as BC metastasis. A 53-year-old woman had been diagnosed with invasive ductal carcinoma (IDC) based on a breast biopsy, and the appendiceal specimen was diagnosed as invasive lobular carcinoma (ILC). We herein report this unique case and provide a detailed review of 13 previous reports.

## Introduction

Breast cancer (BC) is one of the most prevalent types of cancer worldwide [1]. The most common sites of BC metastasis are the lymph nodes, lungs, bone, brain, and liver [2]. However, metastasis of BC to the gastrointestinal tract is uncommon. In particular, metastasis to the appendix is extremely rare, with only 13 case reports in the past 20 years [3].

Appendiceal tumors are frequently identified incidentally during radiological examinations or abdominal surgery, with these procedures often being prompted by tumor-induced acute appendicitis. Accurate preoperative diagnosis is challenging and is typically confirmed by pathological analysis after resection [4].

We herein report a case involving resection of appendiceal metastases of BC and present a review of the relevant literature.

## Case presentation

A 50-year-old woman with no remarkable medical history visited our hospital with the chief complaint of a right breast mass in September 2020. Pathological examination of needle biopsy tissue led to a diagnosis of BC (invasive ductal carcinoma (IDC)). Immunohistochemical examination showed the following: estrogen receptor (ER) (+), progesterone receptor (PgR) (+), and human epidermal growth factor 2 (HER2) (2+). Fluorescence in situ hybridization for HER2 was negative, and the Ki-67 index was low. A computed tomography (CT) scan showed multiple liver metastases, resulting in a diagnosis of stage IV BC. Initially, the patient was treated with leuprorelin and tamoxifen. After one year, new liver metastases prompted a switch to a combination of leuprorelin, fulvestrant, and palbociclib. At two years eight months after the initial treatment, the progression of the liver metastases led to the administration of everolimus plus exemestane as third-line therapy.

**Figure 1:**
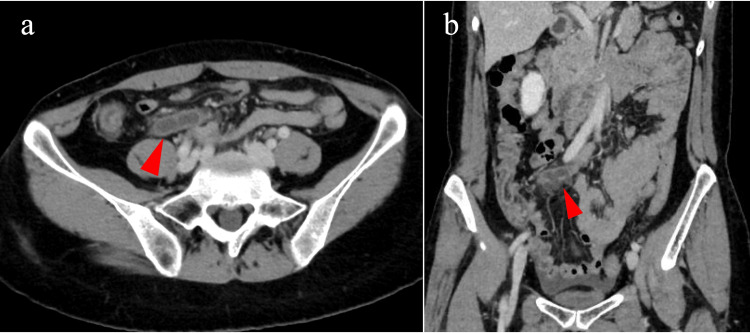
Enlarged appendix (red arrowhead) on contrast-enhanced CT of the abdomen. (a) Axial section. (b) Coronal section. CT, computed tomography

**Figure 2:**
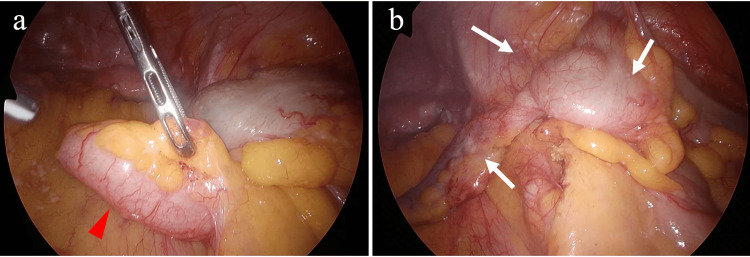
Intraoperative findings. (a) The appendix (red arrowhead) was tense and enlarged. (b) Post-appendiceal mesentery transection. White nodules (white arrows) on the serosal surface of the cecum, appendix, and surrounding peritoneum.

A surveillance CT scan three months after starting the third-line treatment revealed an enlarged appendix (Figure 1). The patient was aware of mild right lower abdominal pain, but a blood test revealed no elevation of the white blood cell count (4700/µL) or C-reactive protein level (0.3 mg/dL). Therefore, an appendiceal tumor was suspected rather than acute appendicitis. Laparoscopic appendectomy was performed at a later date. On laparoscopic observation, the appendix was enlarged in accordance with the preoperative diagnosis, but no ascites was present (Figure 2a). The lower abdominal peritoneum was scattered with small white nodules suspicious of peritoneal dissemination (Figure 2b). Neither biopsy of the nodules nor peritoneal lavage cytology was performed.

**Figure 3:**
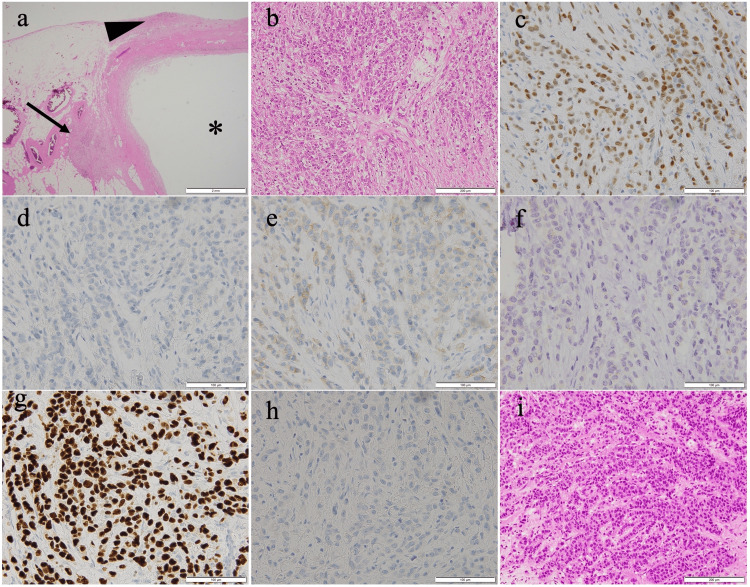
Histopathological and immunohistochemical diagnoses of the appendiceal tumor and breast tumor. (a) H&E image of the appendix at 20×. Tumor cells are seen on the serous surface of the appendix (arrowhead) and in the fat tissue around the appendix (arrow). No tumor is seen on the internal lumen of the appendix (*). (b) H&E image of the appendix at 200×. (c) Immunohistochemical examination of ER. (d) PgR. (e) HER2. (f) E-cadherin. (g) GATA-binding protein 3. (h) Caudal type homeobox 2. (i) H&E image of the tissue from a breast tumor biopsy at the first examination. H&E, hematoxylin-eosin stain; ER, estrogen receptor; PgR, progesterone receptor; HER2, human epidermal growth factor 2

Pathological examination of the appendix showed no primary tumor on the mucosal surface of the appendix, while cancerous cells were found on the serous surface of the appendix and in the surrounding fatty tissue. The immunohistochemical examination revealed ER (+), PgR (−), HER2 (1+), GATA-binding protein 3 (+), caudal type homeobox 2 (−), and E-cadherin (−). The patient was diagnosed with BC metastasis with histological-type invasive lobular carcinoma (ILC) (Figure 3). She was discharged on the third postoperative day with no postoperative complications; the third-line treatment was still being continued five months after the appendectomy.

## Discussion

Past reports have shown that appendiceal metastases from malignant tumors are uncommon, particularly those from BC. In an integrative retrospective study by Connor et al., only 74 (0.9%) of 7970 patients who underwent appendicectomy were found to have appendiceal tumors [5]. Notably, only 11 patients had secondary malignancies, 55% of which originated from primary colorectal cancer. Yoon et al. reviewed 139 patients with secondary appendiceal tumors [6]. The most common origins were the ovary (n=56), colon (n=35), and stomach (n=7), whereas BC was not mentioned.

The route of metastasis to the appendix is considered to involve peritoneal dissemination rather than solitary spread [6]. The mucosal layer is usually intact, whereas progressive invasion of the serosa is frequently observed [7]. This pathological finding was also observed in the present case. Notably, it is very difficult to diagnose peritoneal dissemination without surgery in the absence of ascites or nodules on CT. Therefore, the possibility of a primary appendiceal tumor cannot be ruled out in such patients, even if the patient has a history of other cancers, and the diagnosis is often made as a result of surgery.

As mentioned above, appendiceal metastasis of BC is very rare, and only a few case reports have been published to date. Table 1 summarizes 14 case reports of appendiceal metastatic resections of BC during the past 20 years. IDC was the most common pathological type of BC (76.9%). The hormone receptor status showed a high number of ER-positive cases. Our case was incidentally detected on surveillance CT; however, the lesions in all other reported cases were resected because the patients developed appendicitis. Intraoperative findings of peritoneal dissemination were found in only three (37.5%) of eight cases, including the present case. In a comparison of the pathological type and hormone receptor status of the primary BC and appendix, there were four cases (including the present case) in which the breast was PgR positive whereas the appendix was PgR negative. The hormone receptor status can differ between the primary and recurrent tumors, and hormonal therapy up to the time of appendectomy may affect these findings [8]. In the present case, the BC was diagnosed as IDC, whereas the appendiceal metastasis was diagnosed as ILC. The diagnosis of BC was based on part of the biopsy tissue, not the surgical specimen. It is possible that the tumor originally had an ILC component based on the fact that ILC is able to metastasize to the gastrointestinal tract [9]. Interestingly, Mori et al. reported a similar case in which BC was diagnosed as IDC and the appendix was diagnosed as ILC [10]. By contrast, the histological diagnosis of BC in this case was based on surgical tissue.

**Table 1:** Case reports of appendiceal metastatic resections of BC during the past 20 years. BC, breast cancer; IDC, invasive ductal carcinoma; ILC, invasive lobular carcinoma; ER, estrogen receptor; PgR, progesterone receptor; HER2, human epidermal growth factor 2; CT, computed tomography; NA, not applicable

Authors	Age (y)	Time after cancer diagnosis	Pathology of primary site	ER	PgR	HER2	Basis of diagnosis of appendiceal tumors	Surgical procedure	Peritoneal dissemination	Pathology of appendix	ER	PgR	HER2	Outcome
Varga et al., 2005 [11]	45	NA	IDC	NA	NA	NA	Perforated appendicitis	Open appendectomy	NA	IDC	NA	NA	NA	NA
Pigolkin et al., 2008 [12]	60	18 years	NA	+	+	NA	Acute appendicitis	Open appendectomy	NA	NA	NA	NA	NA	NA
Dirksen et al., 2010 [13]	76	Simultaneous	ILC	NA	NA	NA	Perforated appendicitis	Open appendectomy	Absence	ILC	+	−	−	NA
Mori et al., 2016 [10]	56	2 years 5 months	IDC	+	+	+	Acute appendicitis	Laparoscopic appendectomy	NA	ILC	+	−	+	Well controlled
Kwan et al., 2016 [14]	70	9 months	IDC	+	+	−	Acute appendicitis	Laparoscopic appendectomy	Absence	IDC	+	−	−	Alive 9 months after appendectomy
Araújo et al., 2018 [15]	37	Simultaneous	IDC	NA	NA	NA	Perforated appendicitis	Open appendectomy, hysterectomy, oophorectomy	NA	IDC	−	−	+	NA
Ng et al., 2018 [16]	59	2 years	IDC	−	+	+	Perforated appendicitis	Open right hemicolectomy	NA	IDC	−	+	+	NA
Numan et al., 2019 [17]	44	3 years	ILC	+	+	−	Small bowel obstruction and appendicitis	Open ileocecectomy	Presence	NA	NA	NA	NA	NA
De Pauw et al., 2020 [18]	49	20 years	IDC	+	NA	NA	Acute appendicitis	Laparoscopic appendectomy	Presence	IDC	+	+	−	Died 1 year after appendectomy
Yeola et al., 2021 [19]	59	Simultaneous	IDC	−	−	−	Perforated appendicitis	Open appendectomy	NA	NA	NA	NA	NA	Alive 2 years after appendectomy
Khalil et al., 2022 [20]	59	6 years	IDC	+	+	−	Perforated appendicitis	Open partial cecectomy	Absence	IDC	+	−	−	NA
Hughes et al., 2022 [21]	51	12 years	ILC	+	+	−	Perforated appendicitis	Laparoscopic appendectomy	Absence	ILC	+	+	−	NA
Markovic et al., 2023 [22]	70	9 years	IDC	+	+	−	Acute appendicitis	Open appendectomy	Absence	IDC	+	+	−	Alive 6 months after appendectomy
Present case	53	3 years 1 months	IDC	+	+	−	Surveillance CT	Laparoscopic appendectomy	Presence	ILC	+	−	−	Alive 5 months after appendectomy

## Conclusions

We report a rare case of resected appendiceal metastasis from BC. In this case, the histology differed between the biopsy tissue of the BC and the appendix.

When a patient with a malignant disease presents with an enlarged appendix, the possibility of metastasis should be considered, although it is rare. Furthermore, it should be noted that acute appendicitis can be caused by this appendiceal metastasis, based on previous reports. Recognition of these rare cases and appropriate management are important in treating patients in the complex setting of metastatic BC.
